# Three Mitogen-Activated Protein Kinases Required for Cell Wall Integrity Contribute Greatly to Biocontrol Potential of a Fungal Entomopathogen

**DOI:** 10.1371/journal.pone.0087948

**Published:** 2014-02-03

**Authors:** Ying Chen, Jing Zhu, Sheng-Hua Ying, Ming-Guang Feng

**Affiliations:** Institute of Microbiology, College of Life Sciences, Zhejiang University, Hangzhou, Zhejiang, People's Republic of China; University Paris South, France

## Abstract

Bck1, Mkk1 and Slt2 are three mitogen-activated protein (MAP) kinases constituting cell wall integrity (CWI) pathway that may control multi-stress responses via crosstalk with high-osmolarity glycerol (HOG) pathway in budding yeast. In this study, Bck1, Mkk1 and Slt2 orthologues in *Beauveria bassiana* were confirmed as the three-module cascade essential for CWI because cell wall impairment occurred in the hyphae and conidia of Δ*bck1*, Δ*mkk1* and Δ*slt2* examined in multiple experiments. Strikingly, all the deletion mutants became more sensitive to hyperosmotic NaCl and sorbitol with the Western blot of Hog1 phosphorylation being weakened in Δ*bck1* and absent in Δ*mkk1* and Δ*slt2*. Apart from crossing responses to cell wall perturbation and high osmolarity, three deletion mutants exhibited faster growth and conidiation on nutrition-rich medium, much less virulence to *Galleria mellonella* larvae, and higher sensitivity to nutritional, fungicidal, thermal and UV-B irradiative stresses, accompanied with less accumulation of intracellular mannitol and trehalose. Moreover, Δ*mkk1* and Δ*slt2* were equally more sensitive to all the stresses of different types except wet-heat stress than wild type and more or less different from Δ*bck1* in sensitivity to most of the stresses despite their null responses to two oxidants. All the changes in three deletion mutants were restored by each targeted gene complementation. Taken together, the CWI-required Bck1, Mkk1 and Slt2 are all positive, but differential, regulators of multi-stress tolerance and virulence perhaps due to interplay with the HOG pathway essential for osmoregulation, thereby contributing greatly to the biocontrol potential of the fungal entomopathogen.

## Introduction

Mitogen-activated protein (MAP) kinases in three-module cascades (MAPKKK/MAPKK/MAPK) are a family of serine-threonine proteins which constitute the signaling pathways regulating mating, filamentation growth, high-osmolarity glycerol (HOG), cell wall integrity (CWI), spore wall assembly, and HOG-like Spc1 in eukaryotes [1], [2], [3]. The cascaded kinases can be activated by developmental and environmental cues through the dual phosphorylation of their threonine and tyrosine residues that are functionally conserved [4]. The cascades hallmarked by the MAPKs Slt2 and Hog1 are two stress-responsive pathways mainly responsible for the regulation of CWI and osmotolerance respectively. The CWI-regulated cascade can mediate multi-stress responses via crosstalk with HOG partners in *Saccharomyces cerevisiae* [5], [6], [7]. Functional overlap between the two pathways may also occur in some filamentous fungi. For instance, the Hog1 orthologue Sak1 in *Botrytis cinerea* was phosphorylated upon cell wall challenging and interfered with the phosphorylation status of the Slt2 orthologue Bmp3 [8].

In general, Bck1 (MAPKKK), Mkk1/2 (MAPKK) and Slt2/Mpk1 in the CWI pathway are involved in fortifying cell wall and repairing cell wall damage after stress [1], [4], [9]. Single-gene deletion of the cascaded MAP kinases in *S. cerevisiae* may cause cell autolysis and increased sensitivities to mating pheromone and cell wall perturbing agents, such as Congo red, caffeine and calcofluor white [9]. In human/plant pathogenic fungi, the three MAP kinases are essential not only for CWI but also for conidiation and pathogenicity, as reported with phenotypic changes of their null mutants in *Aspergillus fumigatus* [10], *Coniothyrium minitans* [11], *Cryptococcus neoformans* [12], *Magnaporthe oryzae* [13], and *Ustilago maydis* [14].

CWI is largely determinant to cellular multi-stress tolerance, which is of special importance for fungal insecticides containing unicellular conidia as active ingredients [15]. This is because, upon application, formulated conidia are inevitably exposed to high temperature, solar UV irradiation and applied agrochemicals, which are well known to affect fungal viability, infectivity and thus biocontrol potential [16], [17]. However, the relationship between CWI and multi-stress tolerance has not been fully elucidated in the previous studies on the CWI-regulated MAP kinases of fungal entomopathogens. Recently, Slt2 was found mediating conidiation, hydrophobicity, virulence and tolerance to cell wall perturbation and heat stress in *Beauveria bassiana* [18], an entomopathogenic fungus widely applied to arthropod pest control [16]. Genome analysis has revealed that *B. bassiana* harbors cascaded MAP kinases orthologous to Bck1, Mkk1 and Slt2 in budding yeast [19]. However, it remains unclear whether Bck1 and Mkk1 upstream of Slt2 take equal or differential parts in *B. bassiana* responses to developmental and stressful cues. Nor has an attention been paid to a likelihood of functional overlap between the fungal CWI and HOG pathways. Therefore, this study sought to characterize the functions of Bck1, Mkk1 and Slt2 in *B. bassiana* via multi-phenotypic analyses of their single-gene disruption mutants, and to probe a possible relationship between the CWI and HOG pathways by assessing the effects of the targeted gene disruptions on the phosphorylation of Hog1 under osmotic stress. We found that Slt2 and Mkk1 played equal roles in regulating cellular responses to nutritional, cell wall perturbing, osmotic, fungicidal, and UV-B stresses but to some degree differed from Bck1 in mediating most of the examined stress responses.

## Results

### Features of *bck1*, *mkk1* and *slt2* and their deduced proteins

The coding sequences of *bck1* (4982 bp with two introns), *mkk1* (1776 bp with three introns) and *slt2* (1524 bp with four introns) amplified from the wild-type strain *B. bassiana* ARSEF2860 (Bb2860 or WT herein) encode for the protein sequences of 1622, 538 and 418 amino acids with the predicted molecular sizes of 174.21, 57.81 and 47.56 kDa respectively. The sequences of the deduced Bck1, Mkk1 and Slt2 harbor multi-domains typical for the PKc-like superfamily (Fig. S1 in File S1) and are 43–86%, 54–95% and 67–98% identical to the counterparts of other fungi in NCBI protein database and distinctively classified to fungal Bck1, Mkk1 and Slt2 clades respectively (Fig. S2 in File S1). These implicate that the amplified sequences are the cascaded MAP kinase genes in the CWI pathway of *B. bassiana*.

### Effects of Bck1, Mkk1 and Slt2 on growth, conidiation and viability

**Figure 1:**
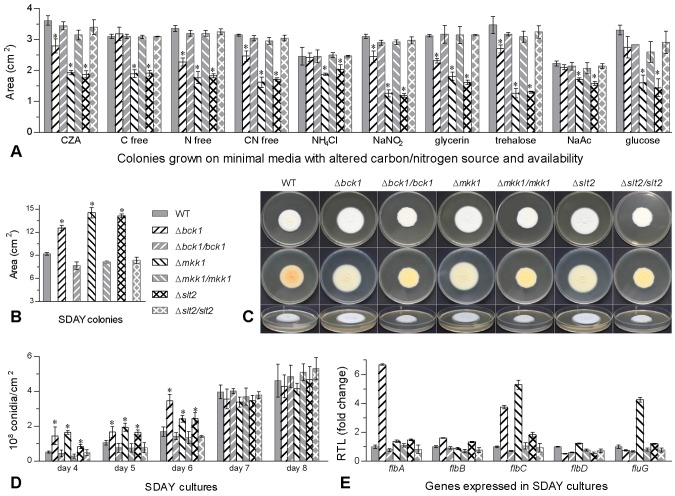
Disruption of *bck1*, *mkk1* or *slt2* in *B. bassiana* affects growth and conidiation. (**A**) Colony sizes after 8-day growth at 25°C on the plates of minimal CZA and CZA-derived media with altered carbon or nitrogen source and availability (free of C, N or both). (**B**, **C**) Sizes and images of fungal colonies after 10-day growth at 25°C on nutrition-rich SDAY plates spotted with 1 µl aliquots of conidial suspension. (**D**) Conidial yields over the days of incubation at 25°C on SDAY plates spread evenly with 100 µl of conidial suspension. (**E**) Relative transcript levels (RTL) of five conidiation-related genes in the cDNAs of mutant strains (versus WT) derived from 3-day SDAY cultures and detected via qRT-PCR with paired primers (Table S2 in File S1). Asterisked bars in each group differ significantly from those unmarked (Tukey's HSD, *P*<0.05). Error bars: SD from three or four (only for E) replicates.

Single-gene disruption/complementation mutants of *bck1*, *mkk1* and *slt2* constructed as described in the Methods section were confirmed by PCR and Southern blot (Fig. S3 in File S1). Compared to parental WT and three complemented mutants (control strains) grown at 25°C, Δ*mkk1* and Δ*slt2* showed similar growth defects on minimal Czapek agar (CZA) and nine CZA-derived media with altered carbon or nitrogen source and availability (Fig. 1A). Their growth defects were worse in most cases than those in Δ*bck1*, which even grew as fast as the control strains on the media with the sole nitrogen or carbon source of NH_4_^+^, glucose or NaAc and with no carbon.

Unlike defective growth on the limited media, the deletion mutants grew significantly faster at 25°C on nutrition-rich SDAY (Sabouraud dextrose agar plus 1% yeast extract), a standard medium for cultivation of entomopathogenic fungi, than the control strains (Fig. 1B) although their colonies were seemingly thinner and more folding (Fig. 1C). After 10-day incubation, colony sizes were averagely increased by 37% for Δ*bck1*, 58% for Δ*mkk1* and 54% for Δ*slt2*. More strikingly, each mutant started conidiation on SDAY much earlier during incubation. Conidial yields measured from the cultures on days 4–6 were increased by 43% to 2.1 fold for the deletion mutants compared to those from the control strains although such differences tended to diminish over the time of incubation and disappeared from then on (Fig. 1D). Five genes associated with conidiation in the mutants were assessed for their transcript levels in 3-day SDAY cultures, in which Bb2860 usually starts formation of conidiophores for conidiation under normal conditions. As a result of quantitative real-time PCR (qRT-PCR), three or four of them were up-regulated by 23% to 4.3-fold in Δ*mkk1*, 60% to 5.7-fold in Δ*bck1*, and 21–83% in Δ*slt2* (Fig. 1E). In contrast, each targeted gene complementation restored their transcripts to normal WT level.

Moreover, conidia from the fresh SDAY cultures were equally viable for all the tested strains due to similar percent germinations within 24 h (*F*_6,14_ = 2.48, *P* = 0.08). However, three deletion mutants lost conidial viability more rapidly than the control strains (*P*<0.0001 in *F* tests) during the storage of their cultures at 4°C. For instance, their 20-day-stored cultures lost 9–13% more viability. After 50-day storage, their cultures had only 4–11% viable conidia, contrasting to ∼44% in the cultures of the control strains. However, microscopic examination of germlings revealed little morphological difference between the deletion mutants and the control strains.

These data indicated that Bck1, Mkk1 and Slt2 negatively regulate the growth and conidiation of *B. bassiana* on SDAY via transcriptional control of at least some conidiation-associated genes but positively the fungal growth on minimal media. The decreased viability seen during culture storage suggests that cell autolysis may be occurring in the deletion cultures although their germlings showed no obvious change in morphology.

### Single-gene disruptions of Bck1, Mkk1 and Slt2 cause cell wall damages

**Figure 2:**
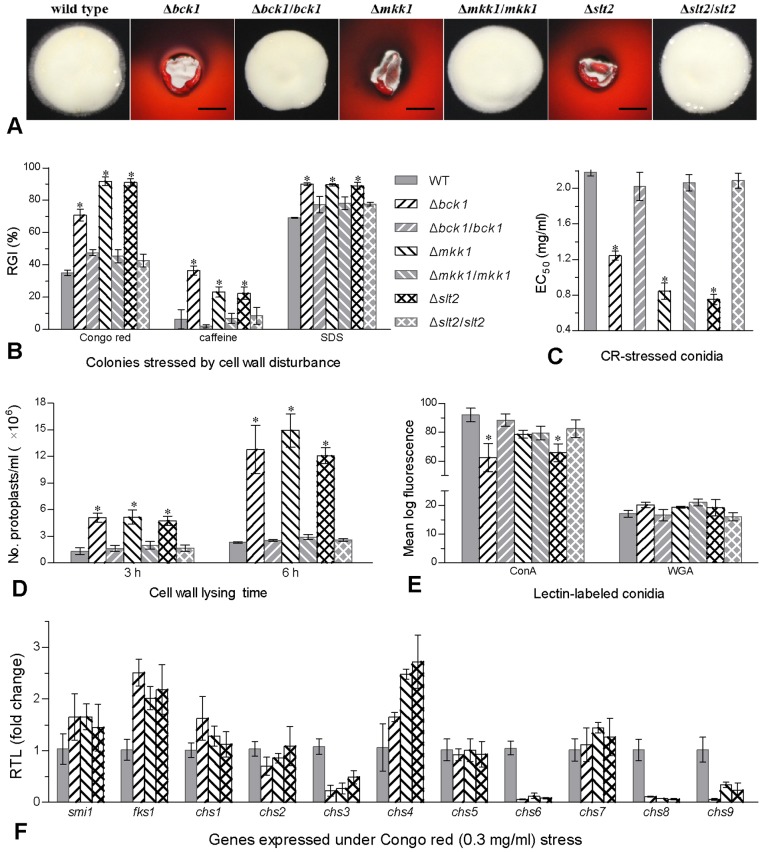
Disruption of *bck1*, *mkk1* or *slt2* in *B. bassiana* causes cell wall impairment. (**A**) Images of fungal colonies grown for 5 days at 25°C on the plates of 1/4 SDAY supplemented with Congo red (1 mg/ml). Scale bars: 5 mm. (**B**) Relative growth inhibition (RGI) of fungal colonies after 5-day growth at 25°C on 1/4 SDAY supplemented with Congo red (1 mg/ml), caffeine (40 µg/ml), SDS (2 mg/ml), respectively. All colonies were initiated by attaching uniform hyphal discs (5 mm diameter). (**C**) Effective concentration (EC_50_) of Congo red (CR) required to inhibit 50% of conidial germination within 24 h at 25°C. (**D**) Counts of protoplasts released from hyphal cells after cell wall lysing of 3 and 6 h with snailase and lysing enzyme (10 mg/ml) under osmotic stress of sorbitol (1.2 M). (**E**) Mean fluorescence intensities detected in 5×10^4^ conidia, labeled with the Alexa Fluor 488-labeled lectins ConA and WGA, via cell flow cytometry. (**F**) Relative transcript levels (RTL) of 11 effector genes in the 1/4 SDAY cultures of three deletion mutants versus WT grown for 3 days at 25°C under the stress of Congo red (0.3 mg/ml). Asterisked bars in each group (B–E) differ significantly from those unmarked (Tukey's HSD, *P*<0.05). Error bars: SD from three repeated assays (B and C), three independent samples (D and E) or four cDNA samples (F) assessed in qRT-PCR with paired primers (Table S2 in File S1).

Single-gene disruptions of *bck1*, *mkk1* and *slt2* resulted in high sensitivity to cell wall perturbation by Congo red, which suppressed or distorted the growth of mutant colonies (Fig. 2A). On 1/4 SDAY supplemented with a sensitive concentration of Congo red, caffeine and SDS, the growth of colonies initiated with small hyphal discs was significantly more inhibited for all the deletion mutants (Tukey's HSD, *P*<0.05) than their control strains (Fig. 2B). Interestingly, Δ*mkk1* and Δ*slt2* exhibited equal sensitivity to each of the cell wall perturbing agents during 5-day incubation at 25°C but differed from Δ*bck1* in sensitivity to Congo red or caffeine. Aside from more inhibited growth, their conidia became highly sensitive to the gradient concentrations of Congo red in germination medium (GM). Modeling analysis of conidial survival trends over the gradients demonstrated that effective concentration (EC_50_) of Congo red required to suppress 50% germination was lowered by 41% for Δ*bck1*, 60% for Δ*mkk1* and 64% for Δ*slt2* compared to the mean (± SD) EC_50_ of 2.09±0.07 mg/ml from the control strains (Fig. 2C).

Aside from the cellular responses to cell wall perturbing agents, cell wall damage was also examined via cell wall degrading assay, in which 10 mg/ml of snailase and lysing enzyme was added to the 3-day Sabouraud dextrose broth (SDB) culture of each strain for cell wall lysing of 3 or 6 h. As a result, the hyphal cells of three deletion mutants released 2.5–2.7-fold and 3.6–5.4-fold more protoplasts than those of the control strains after 3 and 6 h treatment respectively (Fig. 2D).

Possible change of carbohydrate epitopes on conidial surface was probed via lectin binding assay using the fluorescence-labeled lectins ConA [binding specifically to α-glucose and α-N-acetylglucosamine (GlcNAc)] and WGA (binding to β-GlcNAc and sialic acid residues). Mean log fluorescence in 5×10^4^ conidia labeled with ConA decreased by 26% in Δ*bck1* and 22% in Δ*slt2* but insignificantly in Δ*mkk1* (Fig. 2E) while little fluorescence changes were detected in the WGA-labeled conidia of all the tested strains. In addition, four of 11 genes involved in glucan and chitin biosynthesis were transcriptionally down-regulated by 51–94% in Δ*bck1*, Δ*mkk1* and Δ*slt2* under the stress of Congo red (Fig. 2F) based on qRT-PCR with paired primers (Table S2 in File S1). All of them were the coding genes of chitin synthases (*chs3*, *chs6*, *chs8* and *chs9*).

**Figure 3:**
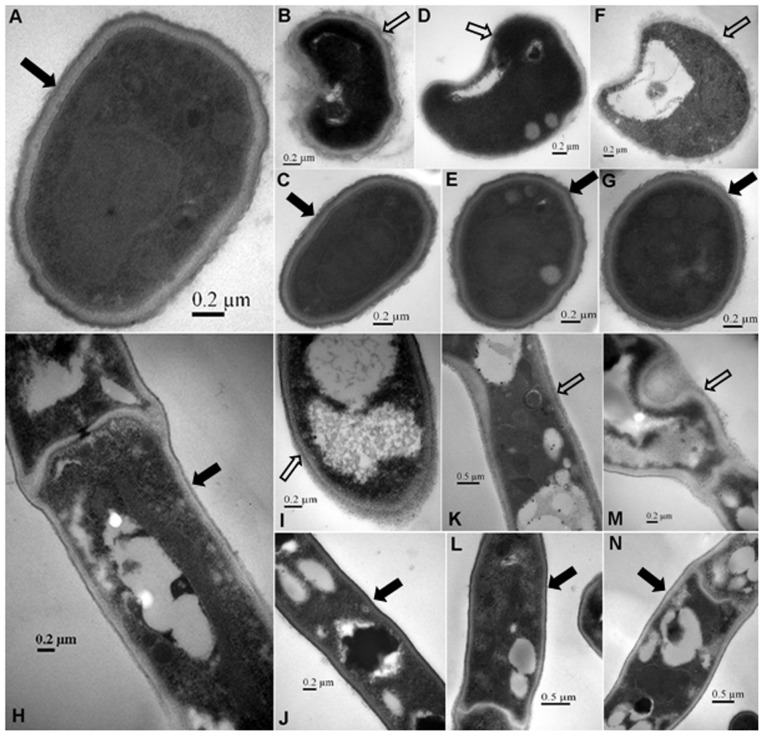
TEM images of *B. bassiana* conidia (A–G) and hyphal cells (H–N). Note the cell wall impairments (open arrows) of conidia and hyphal cells (I, K and M) in Δ*bck1* (B and I), Δ*mkk1* (C and K) and Δ*slt2* (D and M), compared to an outermost electron-dense layer and a distinctly outlined membrane (solid arrows) in the counterpart cells of wild type (A and H) and three targeted gene complementation mutants (C–G, J, L and N).

Furthermore, impaired cell walls of both conidia and hyphal cells were examined via transmission electron microscopy (TEM). As illustrated in Fig. 3A–G, conidia showed an outermost electron-dense layer and a distinctly outlined membrane for all the control strains while conidial walls became thinner or more electron-transparent for all the deletion mutants, making their conidia easily distorted in pre-treatment for TEM. Similar differences in hyphal cell walls were also observed between the deletion mutants and the control strains (Fig. 3H–N).

All the data indicated that Bck1, Mkk1 and Slt2 were the cascaded MAP kinases required for the CWI of *B. bassiana* because their single-gene disruptions resulted in consistently higher cell sensitivity to cell wall perturbation, cell wall damage, and drastic down-regulation of some chitin synthase genes associated with cell wall composition.

### Involvements of Bck1, Mkk1 and Slt2 in osmoregulation

**Figure 4:**
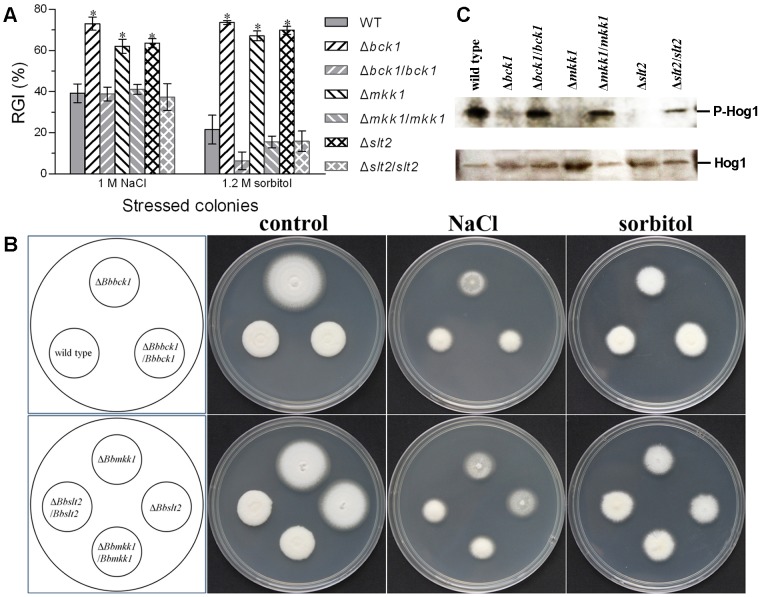
Disruption of *bck1*, *mkk1* and *slt2* in *B. bassiana* inreases osmosensitivity and affects HOG pathway. (**A**) Relative growth inhibition (RGI) of fungal colonies after 8-day growth at 25°C on 1/4 SDAY supplemented with NaCl (1 M) and sorbitol (1.2 M). Asterisked bars in each group differ significantly from those unmarked (Tukey's HSD, *P*<0.05). Error bars: SD from repeated assays of three replicates. (**B**) Images of the fungal colonies grown under osmotic stress. (**C**) Western blots for the existence (lower) and phosphorylation signal (upper) of Hog1 in protein extracts probed with rabbit anti-p38 and anti-phospho-p38 MAPK antibodies respectively. The proteins samples were extracted from the SDB cultures (hyphal cells) of different strains stressed with NaCl (1 M) for 90 min at 25°C.

Apart from the regulation of CWI, Bck1, Mkk1 and Slt2 were also found involving in the osmoregulation of *B. bassiana*. In hyperosmotic assays on 1/4 SDAY plates spotted with 1 µl of conidial suspension, inclusion of NaCl (1 M) in the medium inhibited 1.6–1.9-fold more colony growth of Δ*bck1*, Δ*mkk1* and Δ*slt2* than WT while their growth under the osmotic stress of sorbitol (1.2 M) was even more inhibited by 3.1–3.4 fold (Fig. 4A). Similar situation was also observed on the plates attached with hyphal discs to initiate colony growth under the stresses of both NaCl and sorbitol (data not shown). Notably, Δ*bck1* was more sensitive to either osmotic agent than Δ*mkk1* and Δ*slt2* and each targeted gene complementation restored well the fungal growth to normal WT level.

Due to the increased osmosensitivity, Western blotting analysis was performed to probe the presence and phosphorylation signal of Hog1 in the protein extracts from the SDB cultures of all tested strains stressed with NaCl (1 M) for 90 min using anti-p38 and anti-phospho-p38 MAPK antibodies. As a result, the phosphorylation signal of Hog1 was largely weakened in Δ*bck1* versus the control strains but not blotted in both Δ*mkk1* and Δ*slt2* although the Hog1 protein in their extracts was probed by the anti-p38 antibody (Fig. 4C). Our data indicated that the single-gene disruptions could interfere with the Hog1 phosphorylation of *B. bassiana* and hence increased cellular osmosensitivity. This suggests a likelihood of functional overlap between the fungal CWI and HOG pathways.

### Contributions of Bck1, Mkk1 and Slt2 to biocontrol potential of *B. bassiana*

**Figure 5:**
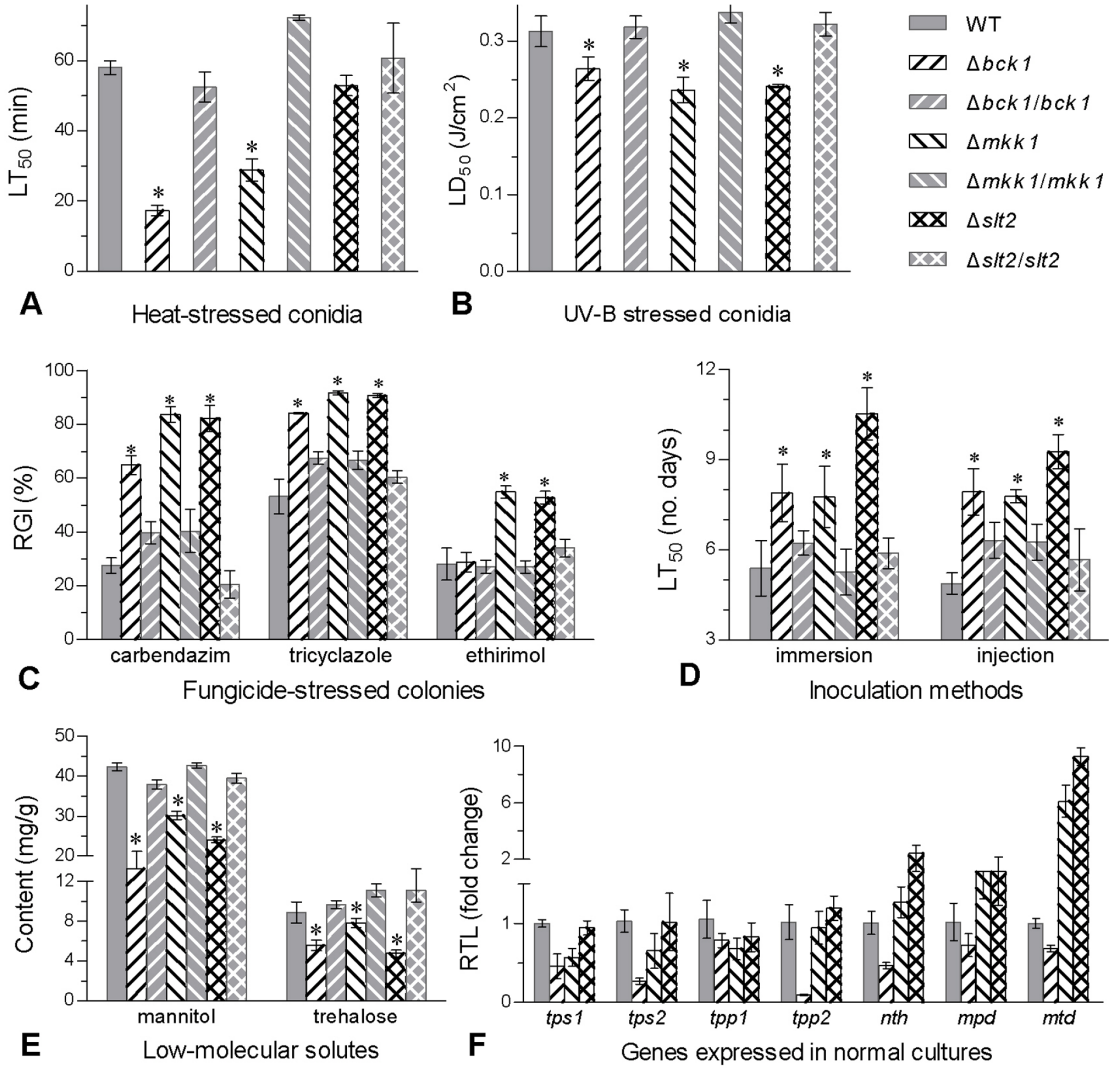
Disruption of *bck1*, *mkk1* or *slt2* reduces the biocontrol potential of *B. bassiana*. (**A**) Medial lethal time (LT_50_) for conidial tolerance to wet-heat stress at 45°C. (**B**) Median lethal dose (LD_50_) for conidial UV-B resistance. (**C**) Relative growth inhibition (RGI) of fungal colonies after 5-day growth at 25°C on 1/4 SDAY supplemented with the fungicides carbendazim (1 µg/ml), tricyclazole (0.2 mg/ml) and ethirimol (0.6 mg/ml). Each colony was initiated by spotting 1 µl of conidial suspension. (**D**) LT_50_ estimates of fungal strains against *G. mellonella* larvae infected by topical application (immersion) and hemocoel injection. (**E**) Contents of mannitol and trehalose (mg/gdry hyphal mass) accumulated in the SDAY cultures grown for 3 days at 25°C. (**F**) Relative transcript levels (RTL) of selected effector genes involved in the biosynthesis and hydrolysis of mannitol and trehalose. Total RNAs were extracted from the 3-day 1/4 SDAY cultures and cDNA samples translated from the total RNAs were assessed for the RTL values of each gene in each deletion mutant versus WT via qRT-PCR with paired primers (Table S2 in File S1). Asterisked bars in each group differ significantly from those unmarked (Tukey's HSD, *P*<0.05). Error bars: SD from repeated assays of three replicates (A–E) or four cDNA samples (F).

Conidial thermotolerance and UV-B resistance were assayed by exposing conidia to the gradient intensities of wet-heat stress at 45°C (0–120 min) and UV-B irradiation (0–0.6 J/cm^2^). Based on their median lethal responses estimated by modeling analysis, conidial thermotolerance decreased by 72% in Δ*bck1* and 53% in Δ*mkk1* but was not significantly affected in Δ*slt2* (Fig. 5A), compared to the mean level from the control strains. Conidial UV-B resistance dropped by 18%, 27% and 25% in Δ*bck1*, Δ*mkk1* and Δ*slt2* (Fig. 5B), respectively. Moreover, Δ*mkk1* and Δ*slt2* were equally more sensitive to the fungicides carbendazim, tricyclazole and ethirimol than the control strains during growth on 1/4 SDAY plates attached with small hyphal discs. After 5-day incubation at 25°C, Δ*mkk1* and Δ*slt2* were ∼51% more sensitive to carbendazim and ∼29% more sensitive to tricyclazole than the control strains while the Δ*bck1* sensitivities to the two fungicides increased only by 33% and 22% respectively (Fig. 5C). In contrast, Δ*bck1* showed null response to ethirimol although this fungicide suppressed significantly the growth of Δ*mkk1* and Δ*slt2* by ∼24%. However, all the tested mutant and WT strains were equally responsive to either H_2_O_2_ or menadione oxidant (*P*>0.05 in one-way ANOVA; data not shown).

In standardized bioassays of *Galleria mellonella* larvae infected by topical application of 10^7^ conidia/ml suspension and hemocoel injection of 500 conidia *per capita*, median lethal time (LT_50_) estimates (Fig. 5D) from time-mortality trends differed significantly among the tested strains [*F*_6,14_ = 16.3 (for cuticle infection) or 16.8 (for hemocoel injection), *P*<0.0001]. Compared to the LT_50_ means of the control strains, the kill actions of cuticle infection and hemocoel injection were slowed down by 39% and 36% in Δ*bck1*, 36% and 35% in Δ*mkk1*, and 85% and 60% in Δ*slt2*, respectively. These virulence defects were attributed to the inactivation of each cascaded component in the CWI pathway of *B. bassiana*.

Taken together, Bck1, Mkk1 and Slt2 regulated positively not only *B. bassiana* tolerance to high temperature, UV-B irradiation and common fungicides but virulence to insect host, thereby contributing greatly to the fungal biocontrol potential.

### Contributions of Bck1, Mkk1 and Slt2 to mannitol and trehalose accumulation

Accumulation levels of intracellular mannitol and trehalose important for fungal anti-stress responses were assessed using hyphal extracts of all the strains grown on SDAY for 3 days at 25°C. As illustrated in Fig. 5E, the contents of mannitol and trehalose were reduced by 51% and 45% in Δ*bck1*, 26% and 23% in Δ*mkk1*, and 41% and 53% in Δ*slt2* compared to the mean (± SD) accumulations of 40.6 (±2.3) and 10.2 (±1.2) mg/g dry hyphal mass in the control strains, respectively.

To gain insight into the reduced mannitol and trehalose contents, seven effector genes involved in the biosynthesis (*mpd*, *tps1*/*2* and *tpp1*/*2*) and hydrolysis (*mtd* and *nth*) of mannitol and trehalose were assessed for their transcripts in the cultures of 1/4 SDAY. As a result of qRT-PCR with paired primers (Table S2 in File S1), the transcript levels of *tps1*/*2*, *tpp1*/*2* and *mpd* in Δ*bck1* were down-regulated by 28–90% while the hydrolysis genes *nth* and *mtd* were up-regulated by 1.3 and 6.1 fold in Δ*mkk1*, and 2.4 and 9.3 fold in Δ*slt2* respectively (Fig. 5F). Higher transcript levels of *nth* and *mtd* relative to those involved in biosynthesis could be partially causative of the lowered mannitol/trehalose accumulation in the deletion mutants.

## Discussion

As presented above, Bck1, Mkk1 and Slt2 are not only essential for the CWI of *B. bassiana* but regulate the fungal growth, asexual development, multi-stress responses and host infection. Their regulative roles are discussed below.

First of all, Bck1, Mkk1 and Slt2 were confirmed as the MAP kinases cascaded for the CWI pathway of *B. bassiana* because their single-gene disruptions caused consistent cell wall damages, as evidenced with increased sensitivity to three cell wall perturbing agents, greatly increased release of protoplasts from hyphal cells treated with cell wall lysing enzymes, and thinner or more electron-transparent cell walls in TEM images. These phenotypic changes in cell wall impairment were similar to those observed in the null mutants of one to three CWI-regulated MAP kinases in other fungi [10], [11], [12], [20], reinforcing a requirement of the Bck1/Mkk1/Slt2 cascade for fungal CWI. Cell wall perturbing agents affect CWI by their binding to cell wall components and thus interfere with cell wall composition. In our study, Δ*bck1* and Δ*slt2* showed some changes of carbohydrate epitopes on the surface of their conidia labeled with ConA. Four chitin synthase genes (*chs3*/*6*/*8*/*9*) in Δ*bck1*, Δ*mkk1* and Δ*slt2* were down-regulated by Congo red, an evidence for the reduced biosynthesis of some chitins as cell wall components. In cell wall-lysing assays, our Δ*bck1*, Δ*mkk1* and Δ*slt2* were hypersensitive to the cell wall-degrading enzymes like the mutants Δ*cpmk2* in *Claviceps purpurea* [21], Δ*MCK1* in *M. oryzae* [13], Δ*Bbslt2* in *B. bassiana* [18], and Δ*CmBck1* and Δ*CmSlt2* in *C. minitans* [11]. Impaired cell walls of our deletion mutants were further evidenced with the TEM images of both conidia and hyphal cells. Therefore, Bck1, Mkk1 and Slt2 in the present study constitute the three-module CWI pathway of *B. bassiana*.

The CWI-regulated Bck1, Mkk1 and Slt2 are all involved in the osmoregulation of *B. bassiana* because their single-gene disruptions resulted in much higher cell sensitivity to NaCl and sorbitol. In Western blotting of protein extracts from hyphal cells treated with hyperosmotic salt, the phosphorylation signal of Hog1 was relatively weak in Δ*bck1* and not probed in Δ*mkk1* and Δ*slt2* with anti-phospho-p38 MAPK antibody although the Hog1 protein in their extracts could be probed by ant-p38 MAPK antibody. These indicate that, in *B. bassiana*, the CWI pathway could functionally overlap, at least in part, with the HOG pathway for osmoregulation although the two stress-responsive pathways are usually conserved [4]. Previously, deletion of yeast *slt2* resulted in a failure of HOG activation under high osmolarity [22], a phenomenon similar to the undetectable phophorylation signal of Hog1 in our Δ*slt2*, while Slt2 in the Hog1-deficient mutant of *Alternaria brassicicola* was inactivated by camalexin [23]. Function loss of *B. cinerea* Hog1 orthologue (Sak1) resulted in lower tolerance to cell wall perturbing agents [8]. The two pathways were also found to co-regulate transcriptional responses to cell wall perturbation by zymolyase [6] and thus to be required for yeast cell survival to cell wall stress [24]. Thus, functional crosstalk between the CWI and HOG pathways is considered existing in yeasts [7] and filamentous fungi [5]. However, it is unclear how the two pathways interplay to regulate cellular multi-stress responses in *B. bassiana* or other fungi. This warrants further studies to reveal direct or indirect protein-protein interactions between Bck1/Mkk1/Slt2 and Hog1 or Hog1-related checkpoints.

Moreover, the growth defects of our deletion mutants on the minimal media were similar to those of previous *B. bassiana* Δ*slt2* [18], *A. fumigates* Δ*bck1*, Δ*mkk2* and Δ*mpkA* [10], *C. minitans* Δ*bck1* and Δ*slt2* [11], and *B. cinerea* Δ*Bmp3* [25]. This highlights a significance of the CWI pathway for *B. bassiana* adaptation to nutritionally limited host integument and habitats. However, our deletion mutants showed faster radial growth on SDAY, a phenomenon different from unaffected growth of single, double and even triple deletion mutants of other fungal CWI-regulated MAP kinases during growth on nutrition-rich YPD [12], [14]. Their conidiation during earlier period of incubation on SDAY was much more abundant than that observed in the control strains. This phenomenon is opposite to severe conidiation defects in the counterparts of *B. bassiana* [18], *B. cinerea* [25], *C. minitans* [11], *M. oryzae* [13] and *C. purpurea* [21]. In addition, the facilitated conidiation of our deletion mutants to some degree was supported by the up-regulated transcription of three or four genes involved in fungal conidiation [26], [27], [28], [29], [30], [31]. Interestingly, conidia in the cultures of our deletion mutants stored at 4°C lost viability more rapidly than those in the control cultures, a phenomenon associated with cell autolysis as seen previously in other fungal counterparts [11], [13], [32], perhaps due to conidial wall damage revealed via cell wall lysing assay and TEM. Thus, Bck1, Mkk1 and Slt2 negatively regulate the growth and conidiation of *B. bassiana* under nutrition-rich conditions but positively contribute to the fungal growth on nutrition-limited substrates.

Furthermore, Bck1, Mkk1 and Slt2 participate in regulating multi-stress tolerance and virulence and hence the biocontrol potential of *B. bassiana*. Interestingly, our Δ*mkk1* and Δ*slt2* were equally sensitive to all nutritional, cell wall perturbing, hyperosmotic, fungicidal and UV-B irradiative stresses but differed from Δ*bck1* in sensitivity to most of the stresses. Exceptionally, conidial thermotolerance was not significantly affected in our Δ*slt2* despite greater decrease in Δ*bck1* than in Δ*mkk1*. More importantly, our deletion mutants killed *G. mellonella* larvae much more slowly than the control strains irrespective of cuticle or cuticle-bypassing infection. This agrees with the severe defect of virulence caused by the deletion of *slt2* in a different *B. bassiana* strain [18], suggesting that the three MAP kinases in the CWI pathway take important parts in regulating the fungal response to host immunity defense during cuticle penetration and after entry into hemocoel. Previously, inactivation of one or two CWI- regulated MAP kinases in phytopathogenic and mycoparasitic fungi also caused severe virulence defects, including *M. oryzae* Δ*bck1* nonpathogenic to susceptible rice seedlings [13], *B. cinerea* Δ*bmp3* less capable of developing necrotic lesions in onion [25], *C. minitans* Δ*bck1* and Δ*slt2* less capable of parasitizing the sclerotia of *Sclerotinia sclerotiorum* [11]. Taken together with our results and those in previous reports, all the CWI-regulated MAP kinases are crucial for fungal responses to chemical, environmental and host cues.

Mechanisms involved in the positive regulation of fungal multi-stress responses and virulence by the CWI-regulated MAP kinases are complicated but likely associated with intracellular and cell-surface events. Apart from the cell wall damages attributed to the single-gene disruptions, intracellular mannitol and trehalose contents were significantly lowered in our deletion mutants, accompanied with altered transcript profiles of several genes associated with the biosynthesis and hydrolysis of mannitol and trehalose. The intracellular solutes are well known as contributors to fungal tolerances to cell wall disturbance, hyperosmolarity, heat shock and UV-B irradiation [33], [34], [35], [36]. Thus, the reduced multi-stress tolerances of our deletion mutants could be attributable to not only cell wall impairment but also less accumulation of intracellular mannitol and trehalose.

In conclusion, Bck1, Mkk1 and Slt2 are required for the CWI of *B. bassiana* and mediate growth and asexual development on nutrition-rich substrate and positively contribute to the fungal virulence and multi-stress tolerance perhaps via interplay with HOG partners. Our results highlight that all the CWI-required MAP kinases are crucial for *B. bassiana* adaptation to various host insects and their diverse habitats, thereby contributing greatly to the fungal biocontrol potential.

## Materials and Methods

### Microbial strains and media

The wild-type strain Bb2860 used as a recipient of target gene manipulation was grown on SDAY (4% glucose, 1% peptone and 1.5% agar plus 1% yeast extracts) at 25°C and 12∶12 h (light:dark cycle). For vector propagation, *Escherichia coli* TOP10 and *E. coli* DH5α from Invitrogen (Shanghai, China) were cultured in Luria-Bertani medium supplemented with kanamycin (100 µg/ml). *Agrobacterium tumefaciens* AGL-1 was cultured in YEB broth [37] at 28°C and used for fungal transformation.

### Cloning and analyses of *bck1*, *mkk1* and *slt2*

The sequences of Bck1, Mkk1 and Slt2 (NCBI codes: EJP69353, EJP70226 and AEU60018 respectively) were blasted from the Bb2860 genome under the NCBI accession ADAH00000000 [19] via BLASTP analysis (http://blast.ncbi.nlm.nih.gov/blast.cgi) using the queries of homologous proteins of several fungi in NCBI protein database. Their full-length gene sequences were amplified from Bb2860 via PCR with paired primers (Table S1 in File S1) and sequenced at Invitrogen for verification. The proteins deduced from the verified sequences were structurally compared with homologues in other fungi, followed by phylogenetic analysis with MEGA5 [38].

### Generation of *bck1*, *mkk1* and *slt2* mutants

Our previous plasmids p0380-bar and p0380-sur-gateway [39], [40] were used as backbones for the manipulation of each target gene. Briefly, the 5′ and 3′ flanking regions of each gene (1692 and 1670 bp for *bck1*, 1655 and 1614 bp for *mkk1*, and 1660 and 1900 bp for *slt2*) were amplified from Bb2860 with paired primers (Table S1 in File S1) and inserted into the corresponding restriction enzyme sides of p0380-bar, yielding the plasmid p0380-*x*up-bar-*x*dn, in which *x* is the target gene *bck1*, *mkk1* or *slt2* and bar is the first marker resistant to phosphinothricin (PPT). To rescue each gene, its full-length nucleotide sequence with flanking regions (8558 bp for *bck1*, 5056 bp for *mkk1* and 5754 bp for *slt2*) were amplified from Bb2860 with paired primers (Table S1 in File S1) and ligated into p0380-sur-gateway to exchange for the gateway fragment with Gateway® BP Clonase™ II Enzyme Mix (Invitrogen), yielding the plasmids p0380-sur-*x*, in which sur is the second marker resistant to chorimuron ethyl.

Each target gene was disrupted by homologous recombination of p0380-*x*up-bar-*x*dn into Bb2860 and rescued by integrating p0380-sur-*x* into the disruption mutant via *Agrobacterium*-mediated transformation [37]. Colonies grown on selective M-100 plates at 25°C were screened in terms of the *bar* resistance to PPT (200 µg/ml) or the *sur* resistance to chorimuron ethyl (10 µg/ml), followed by examination via PCR and Southern blotting with paired primers and with amplified probes (Table S1 in File S1). For Southern blotting, 30 µg of each genomic DNA extracted from the SDAY cultures of putative mutants was digested with *Sac*I (for *bck1*), *Eco*RI/*Hin*dIII (for *mkk1*) or *Spe*I/*Hin*dIII (for *slt2*), separated in 0.7% agarose gel and then transferred to Biodyne B nylon membrane (Gelman Laboratory, Shelton, WA, USA) in Trans-Blot SD Electrophoretic Transfer Cell (Bio-Rad, Hercules, CA, USA). Probe preparation, membrane hybridization and visualization were carried out using DIG High Prime DNA Labeling and Detection Starter Kit II (Roche, Mannheim, Germany). Positive Δ*bck1*, Δ*mkk1* and Δ*slt2* were evaluated together with their parental WT and complemented strains (control strains) for phenotypic changes in triplicate experiments below.

### Assessments of growth rate, conidiation capacity and conidial viability

Three 1 µl aliquots (replicates) of 1×10^7^ conidia/ml suspension of each strain (the same below unless specified otherwise) were spotted centrally onto the plates (9 cm diameter) of SDAY, CZA (3% sucrose, 0.3% NaNO3, 0.05% KCl, 0.1% K_2_HPO_4_, 0.05% MgSO_4_, 0.001% FeSO_4_ and 1.5% agar) and nine CZA-derived media with different carbon/nitrogen sources and availability. The derivatives were prepared by deleting 3% sucrose, 0.3% NaNO_3_ or both, replacing the nitrogen source with 0.3% of NH_4_Cl or NaNO_2_, and using the carbon substitute of 3% glycerin, trehalose, NaAC or glucose respectively. All the plates were incubated for 8 or 10 (only SDAY) days at 25°C and 12∶12 h, followed by cross-measuring colony diameters to compute colony area as a growth index on each medium.

To assess conidiation capacity of each strain, three aliquots of 100 µl conidial suspension were spread onto cellophane-overlaid (CO) SDAY plates at 25°C and 12∶12 h, followed by 7-day incubation at 25°C and 12∶12 h. From day 4 onwards, three culture discs (5 mm diameter) were daily taken from each plate and conidia on each disc was washed off into 1 ml of 0.02% Tween 80 via vortex. The concentration of the conidial suspension was determined using microscopic counts in a hemocytometer and converted to the number of conidia per cm^2^ culture.

The viabilities of the conidia collected from the 7-day cultures and those stored at 4°C for up to 50 days were assessed by spreading three 100 µl suspension aliquots of each strain onto the plates of GM (germination medium: 2% sucrose, 0.5% peptone and 1.5% agar plus 0.02% Tween 80), followed by 24 h incubation at 25°C and 12∶12 h. Percent germination was estimated as an index of conidial viability using three microscopic counts per plate.

### Assaying cell responses to chemical and environmental stresses

Hyphal discs (5 mm diameter) were cut off from 3-day CO-SDAY cultures uniformly initiated by spreading 100 aliquots of conidial suspension and attached centrally to the plates (90 mm diameter) of 1/4 SDAY (1/4 dilutions of SDAY nutrients) alone (control) or supplemented with one of three cell wall perturbing agents [Congo red 1 mg/ml, sodium dodecyl sulfate (SDS) 2 mg/ml and caffeine 40 µg/ml), two oxidants (3 mM menadione and 40 mM H_2_O_2_), two osmotic agents (1 M NaCl and 1.5 M sorbitol) or three fungicides (carbendazim 1 µg/ml, tricyclazole 0.2 mg/ml and ethirimol 0.6 mg/ml). All the plates of three replicates per drug per strain were then incubated for 5 days at 25°C and 12∶12 h. All colony diameters were cross-measured after 5-day incubation. Relative growth inhibition (RGI) of a given stress to each strain was calculated as (*S*_c_−*S*_t_)/(*S*_c_−d)×100, where *S*_c_ and *S*_t_ denote the areas of the unstressed (control) and stressed colonies respectively and d is the constant area of the hyphal disc to initiate each colony.

Since conidia are the active ingredients of common mycoinsecticides, all the strains were assayed for conidial stress responses in repeated experiments. First, conidial tolerance to cell wall disturbance was assayed by adding 100 µl of conidial suspension to 1 ml GB (germination broth, i.e., agar-free GM) supplemented with the gradient concentrations of Congo red (0–4 mg/ml). After 24 h by shaking at 25°C, percent germination was determined with a hemocytometer. Second, conidial tolerance to hyperosmolarity was assayed by spotting 1 µl aliquots of conidial suspension of each strain onto 1/4 SDAY containing 1 M NaCl or 1.2 M sorbitol. After 7-day incubation, RGI was calculated as above. Third, conidial thermotolerance was assayed by exposing 1 ml aliquots of conidial suspension in glass tubes to hot water bath at 45°C for up to 120 min, pipetting 100 µl from each tube every 15 min for 24 h germination in 1 ml GB and assessing percent germination as above. Finally, conidial UV-B resistance was assayed using our previous method [41], [42]. Briefly, 10 µl aliquots of 10^6^ conidia/ml GB were spotted onto central area of glass slides and then exposed to the irradiation of the weighted mean UV-B wavelength of 312 nm (range: 280–320 nm) at the gradient doses of 0–0.6 J/cm^2^ in a Bio-Sun^++^ UV chamber (Viber Lourmat, Marne-la-Vallée, France). After exposure, the slides were incubated at 25°C under saturated humidity for 24 h, stained with cotton blue, and examined for percent germination under a microscope. All the stress assays of each strain were repeated three times. Conidial survival index (*I*_s_) was defined as the ratio of percent germination under a given stress over that in the control. The *I*_s_ trend of each strain over the stress intensities (*x*) was fitted to the equation *I*_s_ = 1/[1+exp(*a*+*bx*)], where *x* denotes the concentration of a stressful chemical, the time length of wet-heat stress or the dose of UV-B irradiation. Solving the fitted equation at *I*_s_ = 0.5 resulted in median lethal intensity for Congo red (EC_50_, mg/ml), wet-heat stress (LT_50_, min) or UV-B (LD_50_, J/cm) to inhibit 50% germination.

### Virulence bioassays

The virulence of each strain was bioassayed on *G. mellonella* larvae (∼300 mg each) using two inoculation methods. For cuticle infection, batches of 30–40 larvae were immersed for 10 s in 30 ml of 10^7^ conidia/ml suspension (treatment) or 0.02% Tween 80 (control), transferred onto towel papers for ∼5 s to remove excessive water and then reared in Petri dishes (15 cm diameter) for up to 13 days at 25°C and 12∶12 h. For cuticle-bypassing infection, each of the batched larvae were injected with 5 µl of 10^5^ conidia/ml suspension (treatment) or 0.02% Tween 80 (control), and then reared at the same regime. Larval mortality in each dish was daily monitored. Time-mortality trends from the bioassays of all the strains repeated three times were differentiated using their median lethal time (LT_50_) estimates, which were generated in probit analysis and used as their virulence indices.

### Observation and assessment of cell wall damage

The method of cell wall degradation [11] was adopted to assess the production of protoplasts from hyphal cells of all fungal strains. For each strain, three 50 ml aliquots (replicates) of 1×10^6^ conidia/ml SDB were shaken at 25°C for 2 days. The resultant hyphal cells were collected via filtration through cellophane film and washed twice with PBS (pH 7.0). Fresh cells of 100 mg from each SDB culture were then resuspended in 2 ml of 1.2 M sorbitol containing snailase and lysing enzyme (Sigma) of 10 mg/ml. After 3 and 6 h incubation at 37°C, cell wall lysing was terminated by keeping the suspensions in ice. Protoplasts released from the hyphal cells of each suspension were examined under microscope and quantified as no. protoplasts/ml using a hemocytometer.

Cell wall damages of conidia from 7-day SDAY cultures and hyphal cells from 3-day SDB cultures were further examined via TEM. Briefly, conidia or hyphal cells were washed three times in PBS, subjected to fixation, dehydration and post-fixation as described previously [43], embedded in Spurr resin, and stained with 2% uranyl acetate and Reynold's lead solution. Ultrathin sections of the treated conidia and hyphal cells were examined for possible cell wall impairment in the TEM model Hitachi H-76520.

Finally, the Alexa Fluor 488-labeled lectins concanavalin A (ConA) and wheat germ agglutinin (WGA) from Molecular Probes-Invitrogen were used to detect possible changes of carbohydrate epitopes on cell surface. Three samples (replicates) of the conidia from the 7-day SDAY culture of each strain were treated following the previous protocol of lectin binding assay [44]. The treated conidia were then reacted with ConA (60 µg/ml) or WGA (20 µg/ml) for 1 h in binding buffer (PBS plus 1 mM CaCl_2_ and 2 mM MnCl_2_ for ConA or plus 1 mM CaCl_2_ for WGA), followed by repeated washing with the binding buffer to remove the lectin residues. Fluorescence signal in 5×10^4^ conidia labeled with each lectin was quantified in cell flow cytometer FC 500 MCL (Becman) using an argon laser at the excitation/emission wavelengths of 488/530 nm. Data acquisition and analysis were performed using CellQuest and FACS Express v3.

### Assessment of intracellular mannitol and trehalose contents

Aliquots (100 µl) of conidial suspension were spread onto CO-SDAY plates, followed by 3-day incubation at 25°C. Three samples (replicates) of 1 g fresh hyphal mass from the culture of each strain were homogenized in liquid nitrogen and suspended in 2 ml dd-H_2_O. The suspension was boiled in water bath for 6 h and centrifuged for 30 min at 16,000 *g*. The supernatant was assayed for the contents of mannitol and trehalose (mg/g dry mass) in an HPLC system using a method described previously [36].

### Transcriptional analyses of selected effector genes

Total RNAs were extracted from the 3-day cultures grown on CO-SDAY (for the expression of conidiation-related genes only) and on 1/4 SDAY alone or supplemented with Congo red (0.3 mg/ml) using RNAiso Plus Kit (TaKaRa, Dalian, China) and translated into cDNAs under the action of PrimeScript® RT reagent Kit (TaKaRa). Four cDNA (20× dilution) samples of each strain were then used as templates to assess the transcript of each gene with SYBR® Premix Ex Taq™ (TaKaRa) via qRT-PCR with paired primers (Table S2 in File S1) using the fungal 18S rRNA as internal standard and the 2^−ΔΔCt^ method [45]. Relative transcript level of each gene was estimated as the transcript ratio of each mutant versus WT.

### Western blotting analysis

Aliquots of 50 ml SDB containing 1×10^6^ conidia/ml were shaken for 3 days at 25°C and then exposed to the osmotic stress of 1 M NaCl for 90 min, followed by collecting hyphal cells via centrifugation. The stressed cells were immediately homogenized in liquid nitrogen and the mixture was suspended in 20 mM PBS (pH 7.0) for protein extraction. The resultant suspension was centrifuged at 15,000 *g* for 30 min at 4°C and the supernatant was assessed for protein concentration with BCA Protein Assay Kit (KeyGen BioTECH, Nanjing, China). Subsequently, aliquots of protein extracts were separated through SDS-PAGE and transferred to polyvinyldene difluoride (PVDF) membranes (Millipore, Germany). Rabbit anti-phospho-p38 MAPK (Thr180/Tyr182) antibody and anti-p38 MAPK antibody (Cell Signaling Technology, Boston, MA, USA) were then used to probe the respective phosphorylation and existence of Hog1 in the samples of each strain following the procedures of Western blot described previously [22]. Bound primary antibodies were revealed using horseradish peroxidase (HRP) conjugated anti-rabbit antibodies and a chemiluminescence detection system (ECL™ Amersham Biosciences).

### Statistical analysis

All phenotypic parameters and fitted estimates from the triplicate experiments were subjected to one-factor (strain) analysis of variance, followed by Tukey's honest significant difference (HSD) test.

## Supporting Information

File S1 **File includes Figures S1–S3 and Tables S1–S2.** Figure S1. Conserved domains located in the Bck1 (A), Mkk1 (B) and Slt2 (C) sequences of *B. bassiana* (Bb2860). Figure S2. Phylogenetic tree constructed for the Bck1 and Mkk1 and Slt2 homologues of *B. bassiana* and selected fungi with MEGA5. The bootstrap values of 1000 replications are given at nodes. Figure S3. Generation and identification of *B*. *bassiana bck1*, *mkk1* and *slt2* mutants. (A–C) Diagrams for the disruptions of *bck1*, *mkk1* and *slt2* respectively. (D–F) The mutants of *bck1*, *mkk1* and *slt2* identified via PCR (*Lanes 1*–*6*) and Southern blotting (*Lanes 7*–*9*) analyses of genomic DNAs with paired primers and amplified probes (Table S1) respectively. *Lanes 1, 4 and 7*: WT. *Lanes 2, 5 and 8*: disruption mutant. *Lanes 3, 6 and 9*: complemented mutant. Table S1. Paired primers designed for the manipulation of *B. bassiana bck1*, *mkk1* and *slt2*. Table S2. Genes and paired primers used in qRT-PCR for assessments of their transcripts in *B. bassiana* cultures under different conditions.
(PDF)
