# Supplementary material for: Three Mitogen-Activated Protein Kinases Required for Cell Wall Integrity Contribute Greatly to Biocontrol Potential of a Fungal Entomopathogen
Source: PLoS One. 2014 Feb 3;9(2):e87948. doi: 10.1371/journal.pone.0087948 (PMC3912201; doi:10.1371/journal.pone.0087948)
Supplement: File S1 — File includes Figures S1–S3 and Tables S1–S2. Figure S1. Conserved domains located in the Bck1 (A), Mkk1 (B) and Slt2 (C) sequences of B. bassiana (Bb2860). Figure S2. Phylogenetic tree constructed for the Bck1 and Mkk1 and Slt2 homologues of B. bassiana and selected fungi with MEGA5. The bootstrap values of 1000 replications are given at nodes. Figure S3. Generation and identification of B. bassiana bck1, mkk1 and slt2 mutants. (A–C) Diagrams for the disruptions of bck1, mkk1 and slt2 respectively. (D–F) The mutants of bck1, mkk1 and slt2 identified via PCR (Lanes 1–6) and Southern blotting (Lanes 7–9) analyses of genomic DNAs with paired primers and amplified probes (Table S1) respectively. Lanes 1, 4 and 7: WT. Lanes 2, 5 and 8: disruption mutant. Lanes 3, 6 and 9: complemented mutant. Table S1. Paired primers designed for the manipulation of B. bassiana bck1, mkk1 and slt2. Table S2. Genes and paired primers used in qRT-PCR for assessments of their transcripts in B. bassiana cultures under different conditions. (PDF) [file pone.0087948.s001.pdf]

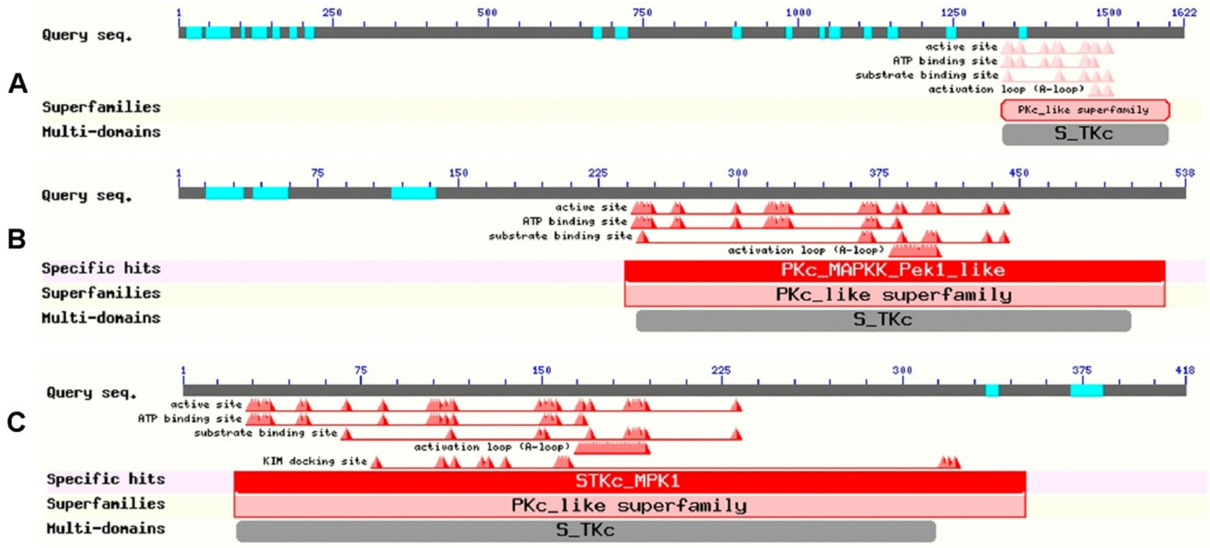

Figure S1. Conserved domains located in the Bck1 (A), Mkk1 (B) and Slt2 (C) sequences of *B. bassiana* (Bb2860).

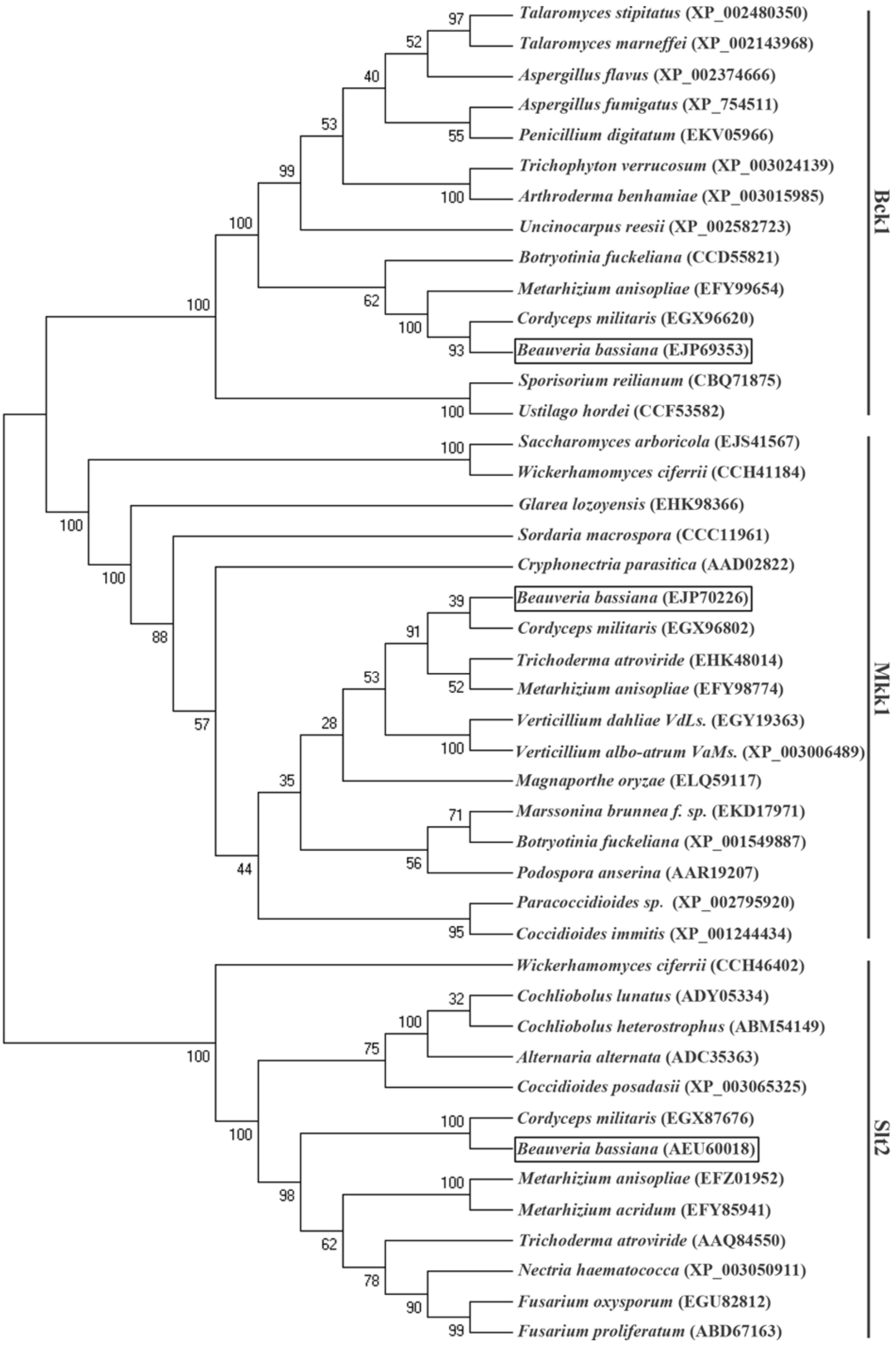

Figure S2. Phylogenic tree constructed for the Bck1 and Mkk1 and Slt2 orthologs of *B. bassiana* and selected fungi with MEGA5. The bootstrap values of 1000 replications are given at nodes.

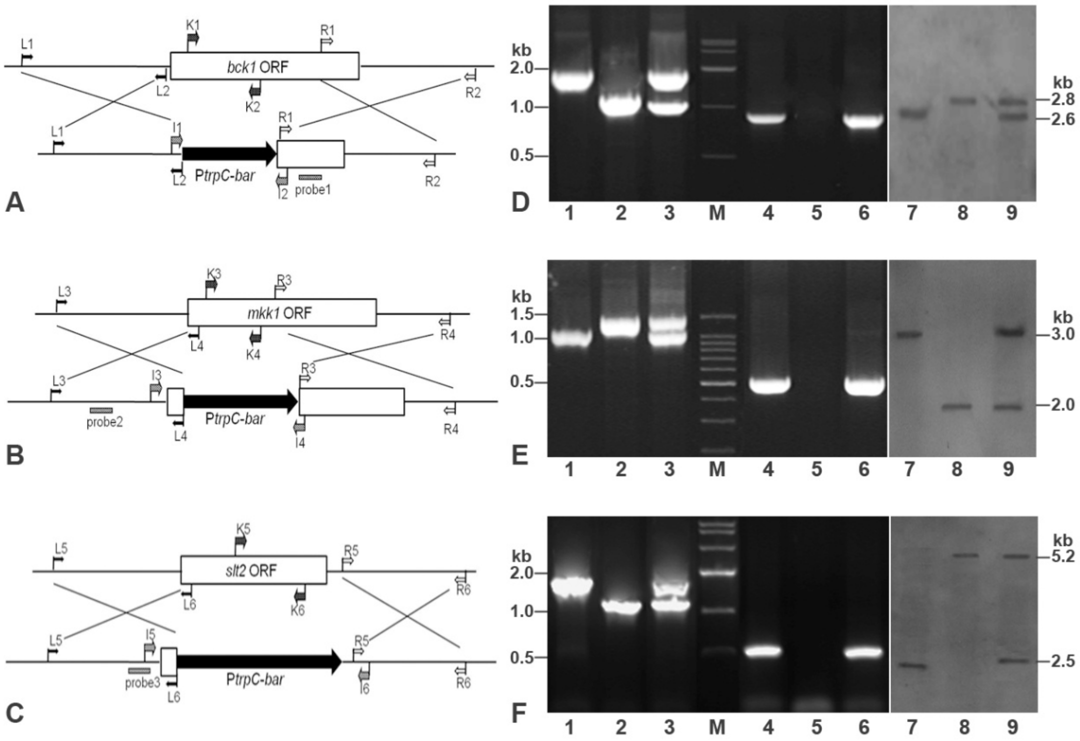

Figure S3. Generation and identification of *B. bassiana bck1*, *mkk1* and *slt2* mutants. (A–C) Diagrams for the disruptions of *bck1*, *mkk1* and *slt2* respectively. (D–F) The mutants of *bck1*, *mkk1* and *slt2* identified via PCR (*Lanes 1–6*) and Southern blotting (*Lanes 7–9*) analyses of genomic DNAs with paired primers and amplified probes (Table S1) respectively. *Lanes 1, 4 and 7*: WT. *Lanes 2, 5 and 8*: disruption mutant. *Lanes 3, 6 and 9*: complemented mutant.

Table S1. Paired primers designed for the manipulation of *B. bassiana bck1*, *mkk1* and *slt2*.

Primers	Paired sequences (5′–3′) *	Purpose
Bck1-F/R	ATGTATCAGGGTGGTCAAAGA / TCACGATTTGTAAGAGTCT	Cloning *bck1*
Mkk1-F/R	ATGGCTGATGCCGGAG / TTAGGCAGCATGAGATGGG	Cloning *mkk1*
Slt2-F/R	ATGGGCGATCTCCAGGGACGCAAAG / CTATCTCATGCTGGCGTCCAGACCA	Cloning *slt2*
qBck1-F/R	ACTTGACCGCTTCGCCTATCG / GGCTGTCTTGAAGGTGGAATTGC	qRT-PCR for *bck1*
qMkk1-F/R	GCAATCATACACCATTACATC / ACTCCAGTAGACATCCATC	qRT-PCR for *mkk1*
qSlt2-F/R	GAGGAGGTGCCCGAGATG / GCTCTGCTGCTGCTGTTG	qRT-PCR for *slt2*
18S-F/R	TGGTTTCTAGGACCGCCGTAA / CCTTGGCAAATGCTTTCGC	qRT-PCR for 18S RNA
L1/L2	AAACCGGAATTCGACCGAGTTTGGCATTCCTTAC / AAACGCGGATCCGCCTCGTCGTAACTGTTTGATG	Cloning *bck1* 5′-end
R1/R2	AACTAGTCTAGAAGCCACTCACCTCACGAATC / AACTAGACTAGTGCCTTACCCTTGTCTTTGT	Cloning *bck1* 3′-end
L3/L4	AAACCGGAATTCATGGGGGTGGTCAAGGTCGTTT / AAACGCGGATCCGTGTTGCTGGAGCGGGAATCTG	Cloning *mlk1* 5′-end
R3/R4	AAACCGCTCGAGTTGGAAATCTCGGTGAAGGTGC / AACTAGTCTAGAGCGCCAAGCTGTATCTGTGAAT	Cloning *mkk1* 3′-end
L5/L6	AAACGCGGATCC ATCACTTGGGGGACTGCTACTT / AAACCCAAGCTT TTGGTCACAGTGTAGCGTTCGT	Cloning *slt2* 5′-end
R5/R6	AACTAGACTAGTGCCGCCATTGCCACGACTTA / AAAAAAGTTAACCTCCTCCACGATGACGAGTAACAG	Cloning *slt2* 3′-end
flBck1-F/R	*GGGGACAAGTTTGTACAAAAAAGCAGGCT*TCTGGTCAAGATTCACTGGAGG / *GGGGACCACTTTGTACAAGAAAGCTGGGT*GCCGATTCATCAGGCTTGTAG	Cloning full-length *bck1*
flMkk1-F/R	*GGGGACAAGTTTGTACAAAAAAGCAGGCT*GACAAGAACTGAGCGTTGGAAG / *GGGGACCACTTTGTACAAGAAAGCTGGGT*ACATACACCGACGACAAGGAGC	Cloning full-length *mkk1*
flSlt2-F/R	*GGGGACAAGTTTGTACAAAAAAGCAGGCT* GACGACTCTACACGCTACACAT / *GGGGACCACTTTGTACAAGAAAGCTGGGT*CTCGCCTCCACTTGCTGCTCT	Cloning full-length *slt2*
I1/I2	ACGGCTGAGACTTCTTGCTTTG / ATCCCCAGATTCGTGAGGTGAG	PCR detecting *bck1*
I3/I4	TTTGTCTCCTTGTCCTACTCAG / GATTGTGGCAGTTGAAGGGTC	PCR detecting *mkk1*
I5/I6	CGTTACTCGTCCATACCTCGGG / GGATTGTCCTCTCTTGGCACTT	PCR detecting *slt2*
K1/K2	TGATCCGACACGTCCATTCCA / CGTCTCATTCGCTTGCCTTCC	PCR detecting *bck1*
K3/K4	CCAGCGTCAAACCTGTCTCTCA / CGACTATGCGTCTTTCCATGCTG	PCR detecting *mkk1*
K5/K6	TAGGCAATGACCACTGTTCGC / CCTGGCTCTGCTGCTGCTGTT	PCR detecting *slt2*
Probe1-F/R	CCCTCTGAGCACCTATTTCCC / CATTAGCGTGAGCGGTCTTTGG	Southern blotting *bck1*
Probe2-F/R	GACCCTTCAACTGCCACAATC / AGACATCCATCTCAGGCTCATC	Southern blotting *mkk1*
Probe3-F/R	GGCAGGCCATACTGGTAATT / TCCACCGTTCTCCATTCTTT	Southern blotting *slt2*

* Underlined regions: the introduced cleavage sites of paired restriction enzymes for disrupting target genes (*bck1*: *Eco*RI/*BamH*I and *Xba*I/ *Spe*I; *mkk1*: *Eco*RI/*Bam*HI and *Xho*I/*Xba*I; *slt2*: *Bam*HI/*Hin*dIII and *Spe*I/*Hpa*I). Italicized and underlined regions: gateway exchange fragments for rescuing the target genes.

Table S2. Genes and paired primers used in qRT-PCR to assess their transcripts in *B. bassiana* cultures under different conditions.

Gene	Tag ID	Annotation	Paired primer sequences (5′–3′) *
**Involved in conidiation**			
flbA	BBA_02968	Regulator of G protein signaling FlbA	CGGATTGGCTGATGGACTGCTC / CCTGGTAGATGGCGTGCTTGG
flbB	BBA_06988	BZIP-type transcription factor FlbB	CACGCTGTTGGACCTGAGTAAAC / CGCCGAATCCGTAACATCTGAC
flbC	BBA_03181	Putative zinc finger protein C	AGACGACACCTCCGCTTGAG / CACTGCTTGCTGCTGTATTGATTG
flbD	BBA_07259	MYB family conidiophore development protein FlbD	CAGCCAGAATCGCCGCAAGAG / AGACGAGCAAGGTGACGGTAGAG
fluG	BBA_04942	FluG protein	GCGAGGAGCAGATTACCGACAG / TCAGGTAGTGACAGATGACTTGGC
**Involved in cell wall integrity**			
smi1	BBA_06704	1,3-beta-glucan biosynthesis protein	AACGGAGGAGATGGTGATG / TCTGATGAGGTCGGATTCG
fks1	BBA_10207	beta-1,3-glucan synthase catalytic subunit	CTCACGCTCACACGCTTCCTC / AGTTGTGGTAGCAGCAAGCAGTAG
chs1	BBA_03793	chitin synthase chaperone-like protein Chs7	GTTCTTCTCGCTCGTTGTTG / CGTGCCGTCCTCATACAG
chs2	BBA_02360	BRCA1 C Terminus domain-containing protein	ATCCTCGGCGTCACCAAG / TCATCCTCGTCCTCGTCATC
chs3	BBA_07346	class VII chitin synthase	TGGTCTACGGCATCTTCAC / CGGTCTGTTGTCCCTCTG
chs4	BBA_06845	MIF4G domain-containing protein	CGCAAGCAGCAATTCGATGTC / GGCAGAGTCAGACGGCAATTC
chs5	BBA_06859	class V chitin synthase	GCTCATACGGCGGCTCTC / CCTTCTTGGTAACGGTCATCAG
chs6	BBA_04667	chitin synthase 3a	CGCTCCCTGCTCGCCCTCTG / GCTTCTTGCCGCCCTTGCCTAAC
chs7	BBA_03590	chitin synthase 1	GGACGGCACAGGCAAGAC / GGTGAGCATCCAGACGAGAAC
chs8	BBA_08396	glycosyltransferase family 2	CGTCGCCGTTTATGAACATC / CTCGCCGTCCTCTTGAATC
chs9	BBA_03236	class2 chitin synthase	CGCTATCGGCTCCACCACATTTG / GCTACTCGCTGCTTCGCTCATC
**Involved in anti-stress responses**			
mpd	BBA_02141	Mannitol-1-phosphate dehydrogenase	ATGCCACTGCCGCCTACC / CGATGCCGTGCTTGCTGAC
mtd	BBA_06629	Mannitol dehydrogenase	CGGCTCGCTCGTCCTCAC / GATGGAGTTGACACGGGCAAAG
nth	BBA_01127	Neutral trehalase	CGCTCCATTCTTGCCGCCATC / TCGTCAAACTCCATGCCATGCTTC
tpp1	BBA_09664	Trehalose-6-phosphate phosphorylase	CGAGCGTCCGTGAAGGTATTAG / CTCCTCCTCCGTTACCGTCAG
tpp2	BBA_08495	Trehalose-6-phosphate phosphorylase	CGATACATACAAGCCAGATG / ACCGAGAACACCTTCAAG
tps1	BBA_02994	Trehalose-6-phosphate synthase	CACCGCCGCCCAGCAAGTC / TCACCGTCACCACCTCTCGTACAG
tps2	BBA_01532	Trehalose-6-phosphate synthase	CGTGTGCCTGGTGTCTTCC / GACTGCCGTTGAGCGACTG

* Gene accession codes in the annotated genome of *B. bassiana* under the NCBI accession ADAH00000000 (Xiao *et al*., 2012).
